# Periorbital Necrotizing Fasciitis: Presentation to management

**DOI:** 10.1016/j.jpra.2025.04.009

**Published:** 2025-04-19

**Authors:** Vladimir Mégevand, Jérôme Martineau, Maria Baccaro, Pauline Darbellay Farhoumand, Daniel F. Kalbermatten, Dominik André-Lévigne

**Affiliations:** a Department of Plastic, Reconstructive, and Aesthetic Surgery, Geneva University Hospitals, Geneva University, 1205, Geneva, Switzerland; b Division of General Internal Medicine, Department of Medicine, Geneva University Hospitals, Geneva, Switzerland

**Keywords:** Eyelid, periorbital, palpebral, necrotizing fasciitis, eyelid reconstruction

## Abstract

Periorbital necrotizing fasciitis is a rare but severe infection that spreads quickly along the fascial planes, often leading to sepsis and is associated with considerable morbidity and high mortality rate. Early recognition is paramount, yet initial symptoms such as localized pain and eyelid swelling are often nonspecific. These are quickly followed by blister formation, periorbital skin and subcutaneous tissue necrosis, and systemic symptoms. Management of necrotizing fasciitis usually involves aggressive surgical debridement alongside broad-spectrum intravenous antibiotics. However, this approach is unsuitable for the periorbital area owing to the risks of eyeball exposure, decline in vision, and disfigurement.

In this report, we present the case of an 85-year-old male patient who was referred to our clinic for evaluation of a suspected upper eyelid abscess, accompanied by a rapidly worsening decline in his overall clinical condition.

The patient underwent 2 surgical debridements of the periorbital area, followed by reconstruction using a combination of a Mustardé cheek advancement flap and full-thickness skin graft. No surgical complications were observed, and the patient achieved aesthetic and functional improvement shortly after definitive reconstruction. Early recognition and debridement are crucial to ensure satisfactory outcomes, while reconstructive surgery techniques allow satisfactory cosmetic results.

## Introduction

Necrotizing fasciitis (NF), commonly referred to as “flesh-eating disease” is a rare but life-threatening infection that affects the skin, muscle, subcutaneous tissues, and underlying fascia. The incidence rate of NF is 4 per 100,000 person-years^1^ and it is characterized by rapidly progressing soft tissue necrosis that invades the superficial and deep fascia and eventually spreads into systemic circulation if not treated immediately.^2^ The causative pathogens are variable and infection is typically polymicrobial; however, monomicrobial infections with group A beta-hemolytic Streptococci (*Streptococcus pyogenes*) or methicillin-resistant *Staphylococcus aureus* (MRSA) have increasingly been reported.^3^^,^^4^ Infection usually develops after triggering events such as trauma, chronic diabetic ulceration, or intravenous drug abuse. Early identification of symptoms is crucial for patient survival, and management often requires one or multiple extensive surgical debridements combined with prolonged polymicrobial antibiotic coverage.^3^

The occurrence of NF in the head and neck area is rare, with most documented cases involving the neck typically resulting from infections originating in the dental or pharyngeal regions.^2^ Facial NF is linked to a high mortality rate and can cause significant facial disfigurement, presenting major challenges for reconstructive surgery. Effective treatment of facial NF requires early detection, swift administration of broad-spectrum antibiotics, surgical debridement to control the infection, and reconstruction of the resulting soft tissue damage.

## Case Presentation

**Figure 1:**
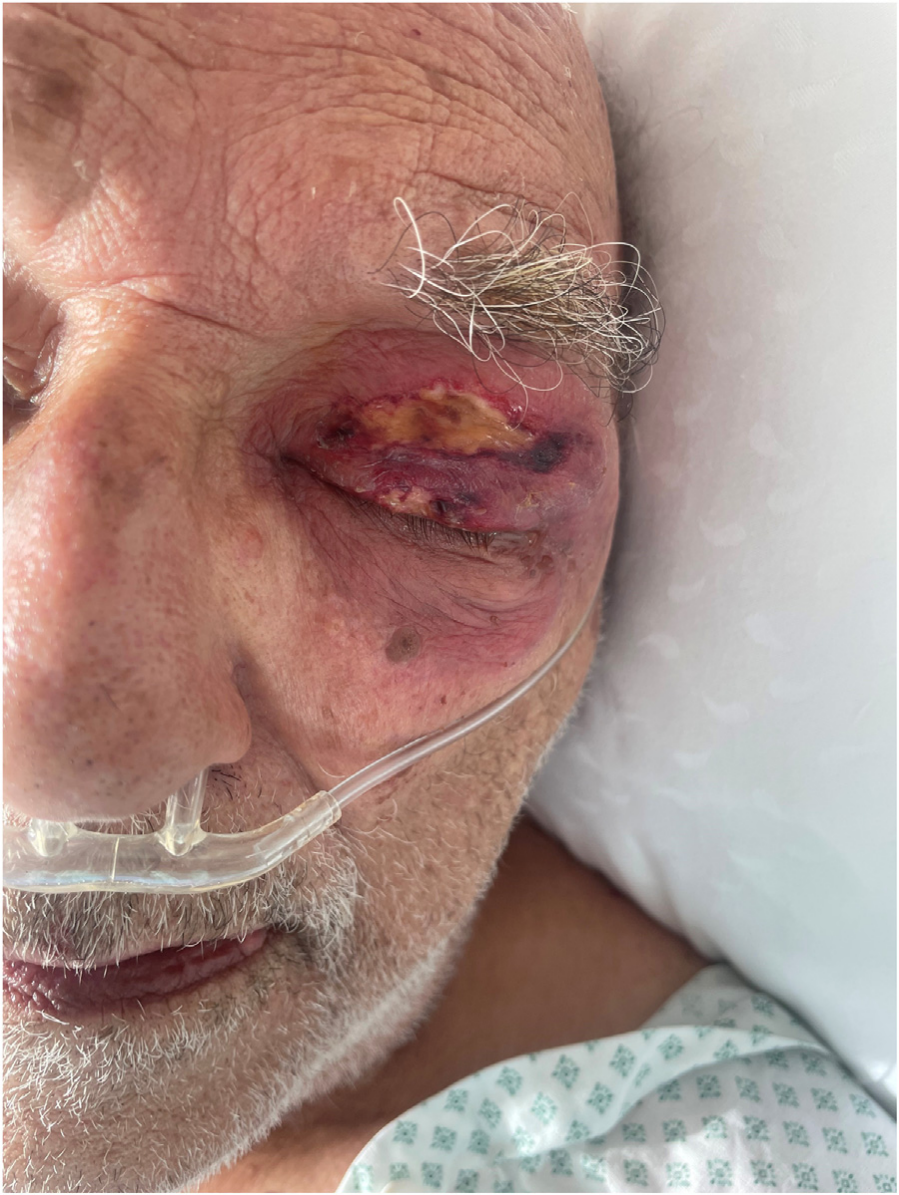
Preoperative assessment

An 85-year-old Caucasian male patient, with a history of arterial hypertension, heart failure, well-controlled type 2 diabetes mellitus, and active smoking status was referred to our plastic surgery clinic in October 2024 with suspected left upper eyelid abscess secondary to a post-traumatic laceration sustained after a fall. He presented with a two-day history of left hemifacial pain and swelling in the periorbital area, associated with erythema and early signs of skin necrosis (Figure 1). Visual acuity was normal. Upon referral, the patient showed signs of sepsis, despite receiving 2 days of intravenous antibiotic therapy. Co-amoxiclav was initiated due to suspected pneumonia, in the context of a temperature of 39.2°C and hypoxemia requiring 4 L/min of oxygen by nasal cannula. Blood workup revealed an inflammatory syndrome, with leukocytosis at 23.6 G/L and core reactive protein at 208 mg/L, as well as a deterioration in renal function (urea 17.3 mmol/l, creatinine 191 µmol/l, and eGFR 29 ml/min/1.73 m^2^). A head and neck CT scan revealed diffuse left periorbital soft tissue infiltration, mainly in the upper eyelid, extending to the preseptal space, orbicularis oculi muscle, temporal fossa, and left masseteric space. No collection or air intensities were detected.

**Figure 2:**
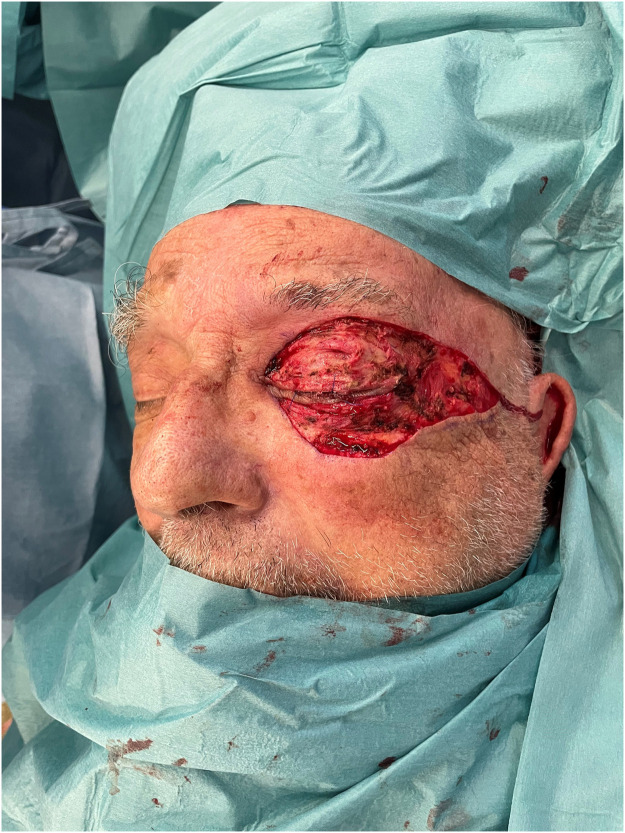
Perioperative status following the first debridement

Given a laboratory risk indicator for necrotizing fasciitis score of 8 and a strong suspicion of NF, the patient was subjected to initial debridement of his upper eyelid under local anesthesia (Figure 2). Bacterial samples and tissue specimens were collected and sent for microbiological and histopathological analysis. Following debridement, a larger spectrum antibiotic regimen, consisting of intravenous vancomycin, imipenem, and clindamycin, was initiated. Blood cultures were also performed as part of the diagnostic workup. The patient was then transferred to a high dependency care unit for close surveillance and management of KDIGO 3 acute kidney failure and hypoxemic respiratory failure, which required high oxygen support (Optiflow 60% FiO2 60 L/min).

Twenty-four hours later, the patient underwent a second surgical debridement under general anesthesia, with the excision of necrotized tissue from the upper and lower eyelids, extending to the left temporal area. His clinical condition improved rapidly.

Bacteriological analysis revealed group A *Streptococcus pyogenes*, and the antibiotic regimen was adjusted to intravenous cefazolin and clindamycin was stopped. Cefazoline therapy was continued for 14 days. Histopathological examination of the superior eyelid and periorbital tissue demonstrated cutaneous ulceration with acute abscessing inflammation, necrotic areas, and involvement of the dermis, hypodermis, and skeletal muscle in the deepest portion of the sampled tissue.

**Figure 3:**
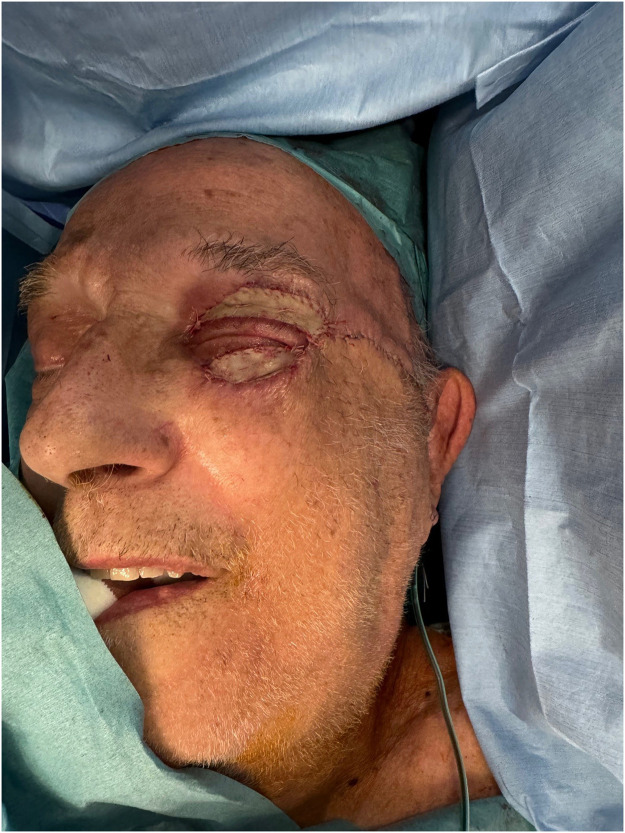
Immediate post-operative status, following reconstruction using a Mustardé cheek advancement flap and full-thickness skin graft

Given the rapid clinical improvement, the patient underwent a definitive reconstruction of his eyelids 2 weeks after the second debridement. We performed a Mustardé cheek advancement flap to reconstruct the lower eyelid. Skin excess from the harvested flap was used as a full-thickness skin graft for upper eyelid reconstruction (Figure 3). A compressive dressing was placed on the skin graft and safely removed on post-operative day 5.

**Figure 4:**
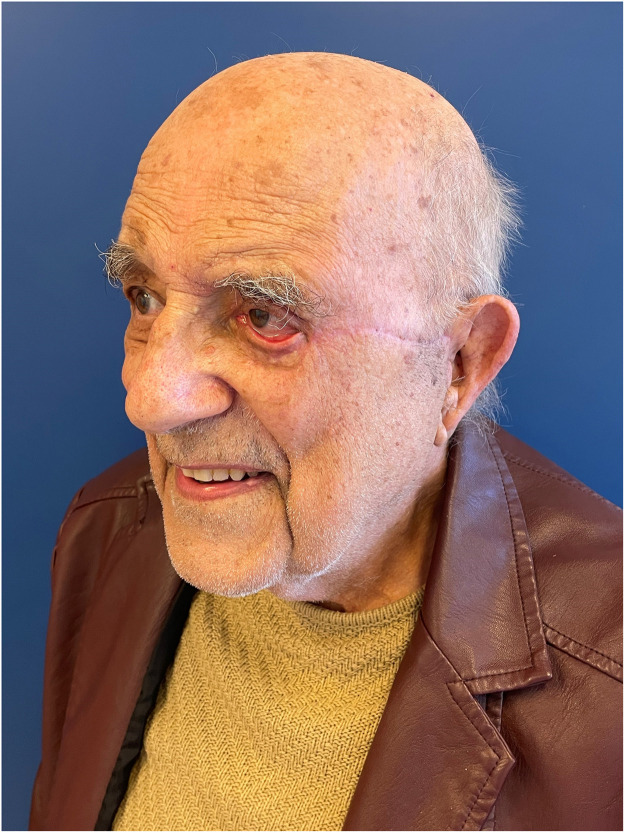
Three-month follow-up

The patient was discharged at one week post-operatively. At the 3-month follow-up, the patient presented with an ectropion of the left lower eyelid (Figure 4), for which lateral tarsal strip was suggested to the patient; however, he refused further surgery. We discharged the patient from our clinic given the satisfactory functional and cosmetic outcomes.

## Discussion

NF is a severe and potentially fatal condition that requires rapid recognition and intervention. This case highlights a rare manifestation of NF in the periorbital region of an older male patient, manifesting as extensive preseptal eyelid cellulitis with periorbital gas and infiltration of the orbicularis oculi muscle. This presentation underscores the importance of considering NF in patients with significant comorbidities, such as diabetes mellitus, in whom infections may progress rapidly. However, with timely diagnosis, surgical intervention, and appropriate antibiotic therapy, a favorable outcome was achieved.

NF is frequently polymicrobial. Therefore, the initiating pathogens may vary depending on the site of origin, often including at least one anaerobic pathogen (commonly Bacteroides spp., Peptostreptococcus spp., and Clostridium spp.), and one or more aerobic species, such as Enterobacteriaceae, Staphylococci, and Streptococci. When a single agent is isolated, it frequently involves a highly virulent pathogen, such as streptococcal and clostridial species. The isolation of group A *Streptococcus pyogenes* (GAS) in our case is consistent with the current literature, where GAS is the most prevalent causative agent of NF^5^ and is increasingly becoming prevalent in cases of periorbital NF.^6^

The rapid progression of NF in the periorbital region can cause substantial morbidity, necessitating swift intervention to prevent systemic spread and significant tissue damage. Periorbital NF appears to be a relatively uncommon presentation of the disease, with few reported cases in the literature, especially with unilateral upper and lower eyelid and orbital tissue involvement, as was observed in our case.^7^

The patient’s age, combined with the underlying chronic conditions such as diabetes, arterial hypertension, and heart failure likely contributed to the rapid and severe progression of the infection. Notably, diabetes is a well-known risk factor for soft tissue infections and poor wound healing due to impaired immune response and microvascular damage.^8^

The role of early surgical debridement in managing NF cannot be overstated. This patient underwent 2 debridement procedures within a short period, which played a pivotal role in halting the spread of infection and facilitating recovery. The use of empirical broad-spectrum antibiotics, which was tailored according to the bacteriological findings, is in line with the recommended practices.^3^

By performing eyelid reconstruction with a Mustardé cheek advancement flap and full-thickness skin grafts 2 weeks after the second debridement, functional and cosmetic outcomes were optimized while minimizing the risk of recurrence. This approach aligns with those in the literature, emphasizing skin grafts as the primary treatment, with or without the use of local flaps.^7^^,^^9^ Even in cases as severe as periorbital NF, successful functional and aesthetic results can be achieved with appropriate surgical management and multidisciplinary care.

## Conclusion

This case reinforces the importance of early recognition, prompt surgical intervention, and tailored antibiotic therapy in the management of NF, particularly when it involves the periorbital area. The favorable outcome achieved in this case emphasizes the potential for recovery, even in the presence of multiple comorbidities, and highlights the need for continued awareness and vigilance in managing complex infections. Further research and documentation of similar cases will help refine the treatment protocols and improve outcomes for patients with such life-threatening condition.

## Conflict of interests

One of the authors is the editor of JPRAS Open and was not involved in the editorial review or the decision to publish this article. All remaining authors declare no conflict of interest.
